# Cholesterol Deficiency Causes Impaired Osmotic Stability of Cultured Red Blood Cells

**DOI:** 10.3389/fphys.2019.01529

**Published:** 2019-12-20

**Authors:** Claudia Bernecker, Harald Köfeler, Georg Pabst, Martin Trötzmüller, Dagmar Kolb, Karl Strohmayer, Slave Trajanoski, Gerhard A. Holzapfel, Peter Schlenke, Isabel Dorn

**Affiliations:** ^1^Department for Blood Group Serology and Transfusion Medicine, Medical University of Graz, Graz, Austria; ^2^Center for Medical Research, Medical University of Graz, Graz, Austria; ^3^Institute of Molecular Biosciences, University of Graz, Biophysics Division, BioTechMed Graz, Graz, Austria; ^4^Division of Cell Biology, Histology and Embryology, Gottfried Schatz Research Center, Medical University of Graz, Graz, Austria; ^5^Institute of Biomechanics, Graz University of Technology, Graz, Austria; ^6^Department of Structural Engineering, Norwegian University of Science and Technology, Trondheim, Norway

**Keywords:** *ex vivo* erythropoiesis, red cell, phospholipids, cholesterol, mass spectrometry

## Abstract

*Ex vivo* generation of red blood cells (cRBCs) is an attractive tool in basic research and for replacing blood components donated by volunteers. As a prerequisite for the survival of cRBCs during storage as well as in the circulation, the quality of the membrane is of utmost importance. Besides the cytoskeleton and embedded proteins, the lipid bilayer is critical for membrane integrity. Although cRBCs suffer from increased fragility, studies investigating the lipid content of their membrane are still lacking. We investigated the membrane lipid profile of cRBCs from CD34^+^ human stem and progenitor cells compared to native red blood cells (nRBCs) and native reticulocytes (nRETs). *Ex vivo* erythropoiesis was performed in a well-established liquid assay. cRBCs showed a maturation grade between nRETs and nRBCs. High-resolution mass spectrometry analysis for cholesterol and the major phospholipid classes, phosphatidylcholine, phosphatidylethanolamine, phosphatidylinositol, phosphatidylserine, sphingomyelin and lysophosphatidylcholin, demonstrated severe cholesterol deficiency in cRBCs. Although cRBCs showed normal deformability capacity, they suffered from increased hemolysis due to minimal changes in the osmotic conditions. After additional lipid supplementation, especially cholesterol during culturing, the cholesterol content of cRBCs increased to a subnormal amount. Concurrently, the osmotic resistance recovered completely and became comparable to that of nRETs. Minor differences in the amount of phospholipids in cRBCs compared to native cells could mainly be attributed to the ongoing membrane remodeling process from the reticulocyte to the erythrocyte stage. Obtained results demonstrate severe cholesterol deficiency as a reason for enhanced fragility of cRBCs. Therefore, the supplementation of lipids, especially cholesterol during *ex vivo* erythropoiesis may overcome this limitation and strengthens the survival of cRBCs *ex vivo* and *in vivo*.

## Introduction

*Ex vivo* generation of red blood cells (RBCs) is a common tool in basic and translational research investigating RBC physiology, RBC affecting disorders and developmental biology. Furthermore, in agreement with overall technical and medical progress, personalized medicine with cultured red blood cells (cRBCs) might become a realistic option for severely alloimmunized patients with chronic blood demand. Current technologies enable the generation of up to 10^6^ cRBCs from a single hematopoietic stem and progenitor cell (HSPC) with homogenous lineage-restricted differentiation and near 100% enucleation (Zeuner et al., 2012; Shah et al., 2014). As an important milestone, the Douays group in 2011 performed the first autologous proof-of-principle transfusion of cRBCs into a human recipient (Giarratana et al., 2011). However, a prerequisite for broader clinical application of cRBCs is the rigorous biological and immunological characterization of these cells. Functional analyses investigating the enzyme content, oxygen uptake and release capacity, and deformability of cRBCs have already been performed (Giarratana et al., 2011). Furthermore, the expression pattern of the most relevant blood group antigens (Fujimi et al., 2008; Giarratana et al., 2011; Hawksworth et al., 2018) and proteome profiles have been reported (Gautier et al., 2016; Wilson et al., 2016). In contrast, little attention has been given to the lipid composition of cRBCs, although changes in membrane lipid organization are critical for the biomechanical stability and longevity of RBCs. Impaired lipid composition might cause the obvious fragility of cRBCs and their limited life span.

The flexible and robust membrane of native red blood cells (nRBCs) consists of a lipid bilayer with embedded proteins attached to the underlying membrane skeleton. In RBCs, cholesterol and a variety of phospholipid species form the core structure of this membrane (Mohandas and Gallagher, 2008). Cholesterol is the most abundant membrane lipid, and due to its single polar hydroxyl group, it is a mainly hydrophobic molecule. Cholesterol is well known to increase hydrocarbon chain order by laterally condensing membrane lipids (Silvius, 2003). This process increases overall membrane rigidity. At the same time, however, cholesterol maintains membrane fluidity. In addition to its lipid-ordering effect, cholesterol is considered to be involved in various processes, including raft formation (Simons and Ikonen, 2000) and specific lipid/protein interactions (Fantini and Barrantes, 2013). Another major membrane lipid is phosphatidylcholine (PC), a glycerophospholipid that creates fluid bilayers when unsaturated. Additional phospholipids in the membrane are phosphatidylethanolamine (PE); phosphatidylinositol (PI), the foundation for phosphoinositides with signaling function; phosphatidylserine (PS); and sphingomyelin (SM), which is the most abundant sphingolipid (van Meer, 2005; Carquin et al., 2014). The various phospholipids differ in their polar head groups and in their esterified non-polar fatty acyl chains.

Interestingly, nRBCs show a high level of specificity with only a few hundred different lipid molecules, although there are thousands of possible combinations. Even interindividual differences in humans are only marginal. Lipids are distributed asymmetrically across the bilayer, which is strictly orchestrated by integral proteins (flippases, floppases, and scramblases). In the RBC steady state, PC and SM are located almost exclusively in the outer leaflet, whereas the inner monolayer contains mostly the amino phospholipids PE and PS with mainly polyunsaturated hydrocarbon chains (Marquardt et al., 2015). In general, the lipid composition is preserved in a narrow range throughout the life span of an erythrocyte. Massive alterations lead to dysfunction and ultimately to cell death. The externalization of internal PS functions as a signal for macrophages to eliminate these cells (Penberthy and Ravichandran, 2016).

The regulation of lipid metabolism during erythropoiesis is poorly understood. Most studies have focused on the lipid content of nRBCs under pathophysiological conditions (Quinn et al., 2009; Wang et al., 2011). In *ex vivo* culture systems, erythroblasts may synthetize their lipids from fatty acids carried by proteins such as albumin or take up lipids from the medium (Migliaccio and Migliaccio, 1987; Huang et al., 2018). Consequently, the lipid pattern of the cells depends on the efficacy of their lipid biosynthesis and the content of the surrounding culture medium (Huang et al., 2018). Some *ex vivo* erythropoiesis models already use different lipid supplementations at various concentrations, while others do not use any (Supplementary Table S1). Currently, a commonly accepted scientific rationale for additional lipid supplementation is still missing. For a comparison of cRBCs and nRBCs, it must be considered that all erythropoiesis models generate mainly reticulocytes (RETs), lacking the final differentiation into biconcave-shaped erythrocytes (Shah et al., 2014). Native reticulocytes (nRETs) released from the bone marrow are irregularly shaped and less flexible than erythrocytes (Malleret et al., 2013). After transendothelial migration into the blood stream, nRETs become functional erythrocytes within 3 days (Chasis et al., 1989). This maturation process includes extensive membrane remodeling, cytoskeletal rearrangement, loss of organelles and RNA and a reduction of the cell volume. Ultimately, the mature erythrocytes are optimized for gas transport and are very flexible and persistent. nRBCs require a high deformation capacity for their frequent passages through the microvasculature and “quality check” in the spleen sinusoids.

To the best of our knowledge, this is the first study to investigate the lipid composition of cRBCs and compare it to that of their native counterparts. The lipid patterns of nRETs, nRBCs and cRBCs were analyzed by high-resolution mass spectrometry. In addition to the seven main lipid classes, 59 phospholipid subtypes were investigated. This quantitative analysis was completed by functional investigation of the membrane deformability and osmotic resistance. Our results demonstrate the importance of lipid, especially cholesterol supplementation during *ex vivo* erythropoiesis and its influence on the functionality of cRBCs.

## Materials and Methods

### Human Specimens and Cell Preparation

CD34^+^ HSPCs were isolated from peripheral blood (PB), (purity 97.8 ± 0.7%) and cord blood (CB) (purity 93.7 ± 2.6%) with magnetic beads as described by the manufacturer (CD34 Microbead Kit Ultrapure, Miltenyi Biotec). nRBCs were obtained from fresh RBC units within 24 h after donation, and nRETs were isolated from CB with magnetic beads within 12 h post-partum (CD71 Microbead Kit; Miltenyi Biotec). Written informed consent was given prior to sampling. The study was approved by the local ethics committee in line with the Declaration of Helsinki (EK 27 165ex 14/15).

### Erythroid Differentiation

CD34^+^ HSPCs from PB and CB were cultured in an established three-step differentiation model in Iscove’s liquid medium (Biochrom) with 5% human plasma (Octapharma) (Giarratana et al., 2011; Betz et al., 2016). This culture medium was used for the generation of cRBC from PB-derived HSPCs (cRBC^pb^) and from CB-derived HSPCs (cRBC^cb^). For lipid-enrichment experiments (generation of cRBC^pb+lipids^ from PB-derived HSPCs), the medium was additionally supplemented with 4 mg/dl cholesterol-rich lipids (Sigma-Aldrich) from day 0 onwards. The lipid content of used media and supplements was measured quantitatively by a clinical chemistry Analyzer AU680 (Beckman Coulter). To obtain the pure enucleated fraction of cRBCs from PB and CB, day 18 cells were filtered through a syringe filter (Acrodisc^®^ WBC Pall). The maturation stage of filtered cells was determined microscopically after New Methylene Blue staining (Reticulocyte Stain, Sigma-Aldrich). Cells were further characterized by flow cytometry on the basis of Thiazole Orange staining (Retic Count^TM^, Becton Dickinson) and expression of CD71. Additionally, the cells were assessed by an ADVIA 212 analyzer (Siemens) for their volume and hemoglobin content. Details are given in the Supplementary Material.

### Lipid Analysis

Lipids were quantitatively measured by mass spectrometry. We analyzed cholesterol, PC (13 subtypes), PE (17 subtypes), PS (2 subtypes), SM (14 subtypes), LPC (7 subtypes), and PI (6 subtypes). Lipids were extracted from cell pellets (10^7^ cells) according to Matyash et al. (2008). Data were acquired according to Triebl et al. (2017) by Orbitrap-MS (LTQ-Orbitrap, Thermo Fisher Scientific). Full-scan spectra from m/z 450 to 1050 for positive ion mode and from m/z 400 to 1000 for negative ion mode were acquired in the Orbitrap mass analyzer at a resolution of 100 k at m/z 400 and <2 ppm mass accuracy. Every sample was measured once in positive polarity and once in negative polarity. For MS/MS experiments, the 10 most abundant ions of the full-scan spectrum were sequentially fragmented in the ion trap using He as the collision gas (CID, normalized collision energy: 50; isolation width: 1.5; activation Q: 0.2; and activation time: 10), and centroided product spectra at a normal scan rate (33 kDa/s) were collected. The exclusion time was set to 10 s. Data analysis was performed by Lipid Data Analyzer (Hartler et al., 2011; Hartler et al., 2017). Details are given in the Supplementary Material.

### Deformability Testing

Filtered cells (4 × 10^8^/ml) were examined on a laser optical rotational cell analyzer (Lorrca^®^; RR Mechatronics Hoorn) according to an established protocol (Plasenzotti et al., 2004; Baskurt et al., 2009). The elongation index (EI) was calculated, describing the deformation of the cells in relation to the applied shear stress.

### Osmotic Resistance

Filtered cells (1 × 10^6^/tube) were incubated in decreasing NaCl concentrations (0.9–0%). To detect free hemoglobin, 50 μl of supernatant was analyzed in 500 μl of Harboe buffer (Bioanalytic) with the Harboe 3-wavelength-absorption method (380, 415, 450 nm) using a Shimadzu-1800 spectrophotometer (Harboe, 1959).

### Cholesterol Depletion

Cholesterol depletion was performed using Methyl-β-cyclodextrin (MBCD, Sigma-Aldrich) as previously described (Domingues et al., 2010). nRBCs were obtained from fresh RBC units and suspended up to 20% hematocrit in 5 and 6.5 mM MBCD in PBS buffer. Cells were incubated 30 min at 37°C and then washed three times with PBS by centrifugation at 500 *g* for 10 min to remove the MBCD-cholesterol complexes. Cholesterol-depleted cells were analyzed for osmotic resistance as described above. Non-treated RBCs were used as controls.

### Statistics

The non-parametric Mann–Whitney *U* test and Kruskal–Wallis test with subsequent Bonferroni correction were performed to test for differences between groups. *p* < 0.05 was considered statistically significant.

## Results

### Erythroid Differentiation

**FIGURE 1:**
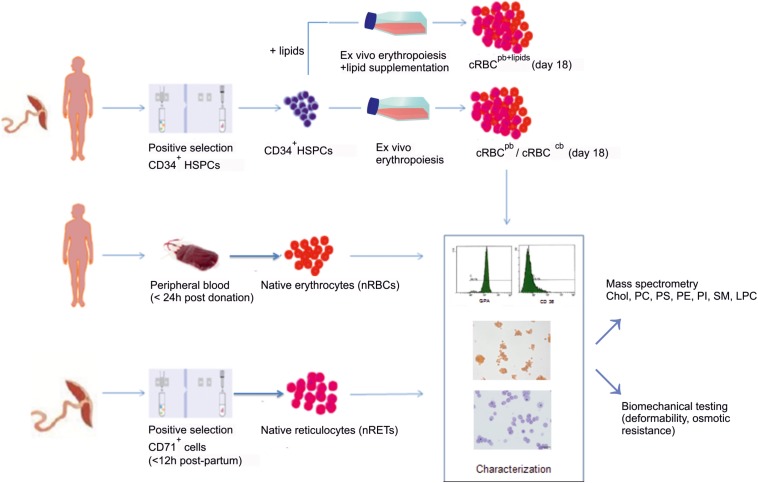
Overall scheme of the study. In an initial set of experiments, *ex vivo* erythropoiesis was performed from CD34^+^HSPCs derived from PB (cRBC^pb^) and CB (cRBC^cb^) in an established liquid culture system. Filtered, enucleated day 18 cells were compared to PB-derived nRBCs and CB-derived nRETs. Cells from all sources were characterized by flow cytometry and immunohistochemistry regarding their maturation stage. The cellular cholesterol and phospholipid contents were measured by mass spectrometry. In addition, the deformability and osmotic resistance of cells were analyzed. Based on results from initial experiments, we performed additional experiments from CD34^+^ HSPCs by adding cholesterol rich lipids to the culture medium (cRBC^pb+lipids^).

For extensive lipid analysis of cRBCs, *ex vivo* erythropoiesis from CD34^+^ PB HSPCs (cRBC^pb^) and CD34^+^ CB HSPCs (cRBC^cb^) was performed in a well-established three-phase erythropoiesis assay (Giarratana et al., 2011; Betz et al., 2016). An overview of the study is given in Figure 1. The used culture medium for the generation of cRBC^pb^ and cRBC^cb^ contained 5% human plasma as the only lipid source. The complete culture medium contained 3 mg/dl cholesterol, 5 mg/dl triglycerides and 13 mg/dl phospholipids. Lipid concentrations of used media and supplements are summarized in Supplementary Table S2. The homogeneous differentiation of CD34^+^ HSPCs into terminally mature erythroid cells was confirmed by morphology and flow cytometry (Supplementary Figure S1) (Giarratana et al., 2011; Betz et al., 2016). The cumulative expansion averaged 0.5 × 10^5^ ± 0.2 × 10^5^-fold and 1.1 × 10^5^ ± 0.1 × 10^5^-fold in PB-/CB-derived cultures. Compared to cRBC^pb^, cRBC^cb^ showed a slightly delayed maturation as indicated by a lower percentage of hemoglobin^+^ cells on days 8 and 11 followed by a delay in further enucleation (Supplementary Figures S1D,H). This observation was also reflected by cell surface marker expression. Although already on day 8 >95% of cells from both sources expressed the early erythroid marker CD36, in cRBC^cb^, higher percentages of CD45^+^ erythroblasts and lower percentages of already glycophorin A^+^ (GPA^+^) cells were observed on days 8 and 11 (Supplementary Figures S1C,G). On final culture days >99% of cells from both sources expressed the erythroid marker GPA. In line with terminal maturation, the percentage of CD36^+^ cells decreased to 21.4 ± 7.0% in cRBC^pb^ and 59.2 ± 12.5% in cRBC^cb^ (Kieffer et al., 1989; Hu et al., 2013). The enucleation reached 83.0 ± 7.0% and 70.0 ± 6.0% for cRBC^pb^ and cRBC^cb^, respectively. To eliminate the remaining nucleated normoblasts and extruded nuclei, on day 18 a filtration step was performed. The obtained purity of enucleated cells was >98% for both sources, ensuring comparability for subsequent lipid profiling.

### Maturation Stage of cRBCs

**FIGURE 2:**
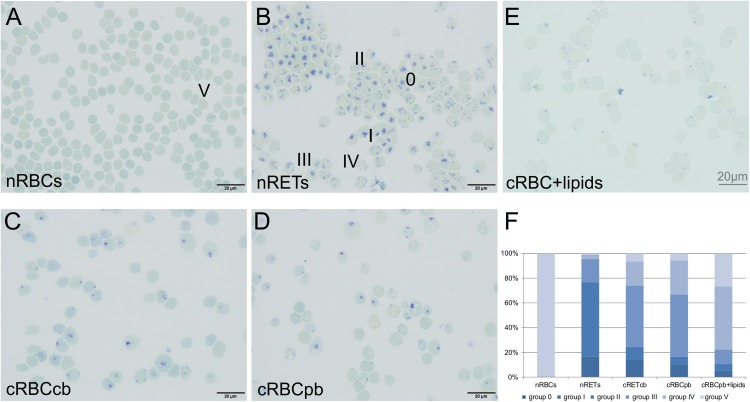
Maturation stage of nRBCs, nRETs, cRBC^cb^, cRBC^pb^ and cRBC^pb+lipids^ (filtered day 18 cells) determined by microscopic evaluation after New Methylene Blue staining. **(A–E)** Representative pictures of cells from all sources. Bright field, oil, 100 × magnification (scale bar: 20 μm). Dependent on the remaining RNA network, cells are classified into stages 0 to V. Stage 0 shows the typical reticular mesh-like structures of immature cells stained in dark blue, while stage V represents fully mature erythrocytes without any dark blue reticular staining. **(F)** Diagram showing the affiliation of cells for the different maturation stages in nRBCs, nRETs, cRBC^cb^,cRBC^pb^ and cRBC^pb+lipids^ (*n* = 4, each) displayed in%.

The maturation stage of filtered cRBCs was determined by New Methylene Blue staining. Depending on the content of remaining ribosomal organelles, maturation stages were classified between 0 (dense chromatin residues) and V (no chromatin residues) (Figure 2; Koepke and Koepke, 1986; Houwen, 1992). nRETs isolated from CB samples by CD71 purification (purity >98%) showed an almost homogeneous maturation stage of young nRETs with typical dark blue mesh-like ribosomal RNA residuals, corresponding to stage 0-II. nRBCs lacked chromatin spots, corresponding to stage V. In comparison to these controls, cRBCs showed a mixture of all five stages, displaying an intermediate maturation between nRETs and nRBCs. A comparable maturation pattern of cRBC^pb^ was obtained by flow cytometry after Thiazole Orange staining for the detection of remaining nucleic acids together with CD71 (Supplementary Figure S2). 74.4 ± 6.9% of the cRBC^pb^ stained positive for Thiazole Orange, compared to 88.0 ± 4.2% of nRETs and only 2.6 ± 0.6% of nRBCs. In line with an intermediate maturation stage of cRBCs, they also differed from nRETs and nRBCs in the expression of the Transferrin receptor CD71, which is known to be downregulated during terminal reticulocyte maturation (Supplementary Figures S1, S2) (Hu et al., 2013; Malleret et al., 2013). Based on New methylene Blue staining, cRBC^pb^ reached a slightly higher maturation grade than did cRBC^cb^. This slight difference between cRBC^pb^ and cRBC^cb^ became also evident by higher cell surface expression of CD71 and CD36 in cRBC^cb^ (Supplementary Figures S1C,G). Additionally, the mean corpuscular volume (MCV), mean corpuscular hemoglobin content (CHm) and mean corpuscular hemoglobin concentration (CHCm) of cRBC^pb^ were analyzed by ADVIA analyzer (Supplementary Table S3). Under *ex vivo* conditions, cRBC^pb^ had an increased MCV (141.5 ± 9.7 fl) compared to that of nRET^pb^ (103.5 ± 3.0 fl), which is in line with stress-induced erythropoiesis, as previously reported (Giarratana et al., 2011). CHCm was reduced (25.5 ± 6.0 g/dl) compared to our own data for nRET^pb^ (31.7 ± 1.1 g/dl), although near the normal range published for PB-derived reticulocytes measured by ADVIA analyzer (27–33 g/dl) (Nebe et al., 2011).

### Lipid Composition of cRBCs

**FIGURE 3:**
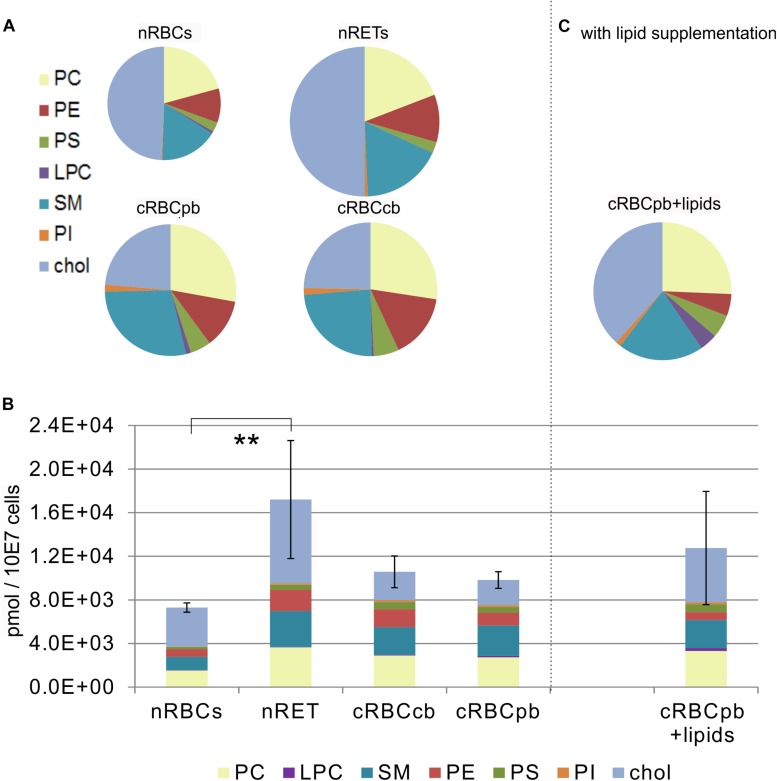
Lipid content measured by high-resolution mass spectrometry in nRBCs, nRETs, cRBC^pb^, cRBC^cb^ and cRBC after lipid supplementation (cRBC^pb+lipids^) (*n* = 4 each) **(A)** Proportional lipid content including PE, PC, PS, PI, SM, LPC and cholesterol of nRBCs, nRETs, cRBC^pb^, cRBC^cb^ and **(C)** cRBC^pb+lipids^ displayed as pie charts. The magnitudes of the circles correspond to the absolute lipid content of the cell type. **(B)** Absolute lipid content of nRBCs, nRETs, cRBC^pb^ and cRBC^cb^ and cRBC^pb+lipids^ (*n* = 4 each) regarding the phospholipid groups PC, PE, PS, PI, SM, LPC, and cholesterol. Values are depicted as pmol/10^7^ cells (^∗∗^*p* < 0.01).

**FIGURE 4:**
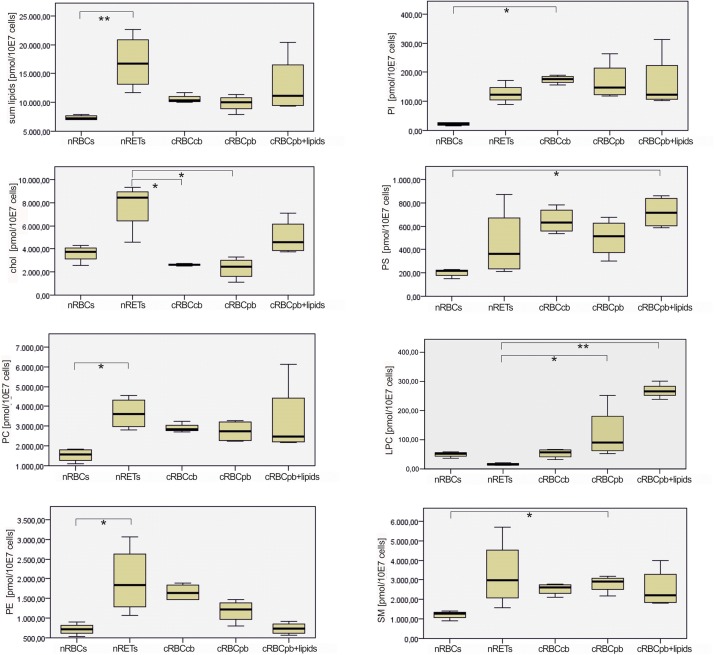
Box and Whisker Blots showing differences in absolute contents of cholesterol and measured phospholipid species between nRBCs, nRETs, cRBC^cb^, cRBC^pb^, and cRBC^pb+lipids^ (*n* = 4, each). Samples were analyzed with Kruskal–Wallis Test for independent samples. Significance values were adjusted with Bonferroni correction (^∗^*p* < 0.05; ^∗∗^*p* < 0.01).

Quantitative comparison of the lipid content of RBCs (cholesterol, PC, PS, SM, PE, PI and lysophosphatidylcholine (LPC) was performed by high-resolution mass spectrometry. In nRBCs, cholesterol represented the largest fraction, with 49.2 ± 9.9% of the total lipid content, followed by PC (20.7 ± 4.3%) and SM (16.6 ± 3.1%). The sum of all the other lipids constituted the remaining 13.5 ± 3.1% (Figure 3A and Supplementary Figure S3). Interestingly, exactly the same pattern was found in nRETs, although the absolute content of lipids (17.2 ± 5.4 nmol/10^7^ cells) was significantly higher than that in nRBCs (7.3 ± 0.4 nmol/10^7^ cells), in line with the larger cell size and membrane surface (Figure 3B). In more detail, in nRETs higher absolute amounts were observed for each individual lipid type with the exception of LPC, reaching statistical significance for PC and PE (Figure 4).

With a mean total lipid content of 10.2 ± 0.5 nmol/10^7^ cells, the lipid content of cRBCs was between that of the native sources. In comparison to native cells, the cholesterol content was obviously impaired in cRBCs, indicating a severe culture artifact. This phenomenon was reflected by both the absolute (2.3 ± 0.9 nmol/10^7^ cells) and relative (23.7 ± 8.1%) cholesterol contents (Figures 3, 4 and Supplementary Figure S3). Despite some minor variations, we did not observe any significant differences in phospholipids between cRBCs derived from CB and PB. These minor variations might reflect differences of the biological replicates or the slightly lower maturation stage of cRBC^cb^ as described above. However, only cRBC^cb^ differed in their higher absolute amount of PI from nRBCs and only cRBC^pb^ in their higher absolute amount of SM from nRBCs (Figure 4). Observed minor variations between both sources might be the reason for these different significance levels compared to nRBCs. In general, the content of PI and SM was comparable between cRBC^pb^ and cRBC^cb^ and like in nRETs higher than in nRBCs. Interestingly, only in nRBCs was the total amount of LPC higher than that of PI, while in nRETs and cRBCs, the PI content was higher. The highest LPC content was observed in cRBC^pb^. Additional calculations of the average chain lengths and double bonds of the phospholipid classes are given in the Supplementary Material and Supplementary Figures S4, S5. While a longer lipid chain causes lower flexibility, a higher number of double bonds accounts for higher flexibility of the molecule.

### Lipid Subtype Distribution in cRBCs

**FIGURE 5:**
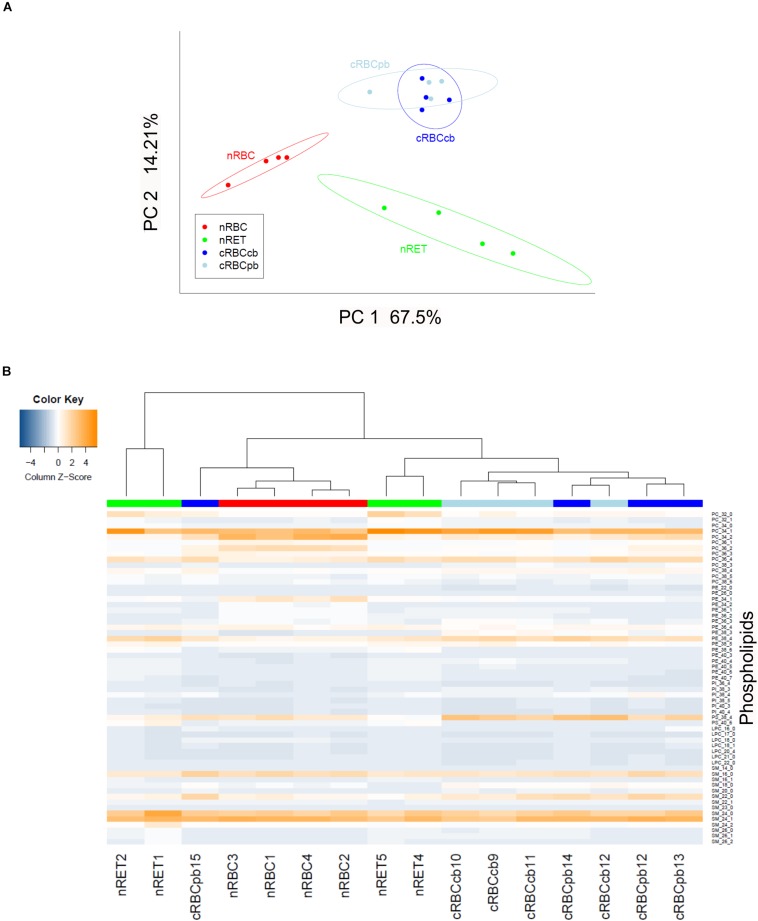
Statistical evaluation of lipidomics data after lipid subtype analysis. **(A)** Principle component analysis showing clustering of nRETs, nRBCs, cRBC^pb^, and cRBC^cb^ (*n* = 4 each). The ellipses show the 95% confidence interval. The first two principal components (PC1 and PC2) covered 81.71% of the data variability. **(B)** Heat map with hierarchical clustering analysis. Color bars mark the different sources (red: nRBCs, green: nRETs, dark blue: cRBC^pb^, light blue: cRBC^cb^). To standardize the data for the heatmap, z-scores over the samples were calculated by subtracting the sample mean and dividing over its standard deviation. Coloring is helping to easily visualize whole data, where blue tones show lower values and orange tones higher values. The reader can immediately recognize data patterns as well as similarities/differences between samples. The color key of the heat map shows the column z-scores. Cell types were clustered with the complete linkage agglomerative hierarchical clustering method on Euclidean distances. We used this statistical method to group the samples according to their phospholipid profiles.

Next, we performed a detailed analysis of a total number of 59 phospholipid subtypes. The statistical evaluation of lipidomics data showed a distinct clustering of cRBC^cb^/cRBC^pb^ versus nRETs and nRBCs in principal component analysis (PCoA) (Figure 5A). In addition to the PCoA analysis, phospholipid subtype profiles are presented in a heatmap, where the contents of individual lipid subclasses according to the standardized *Z*-score values are represented by different colors (Figure 5B). Hierarchical clustering was performed to group the samples according to their phospholipid profiles. The nRBCs (red label) show a homogenous pattern in the heat map and close relatedness in the hierarchical clustering tree above, revealing a homogenous group of samples. nRETs (green label) are clustering apart from the nRBCs and show some inhomogeneity among their group. This might be caused by their different biological origins and maturity levels. The two cRBC groups (light blue and dark blue labels) are clustering as one group closely related to nRETs. Only one exception (cRBC^pb^ dark blue) is clustering with nRBCs. The reason for this outlier remains elusive so far. These findings were strengthened by the correlation analysis, as shown in Supplementary Figure S6.

**FIGURE 6:**
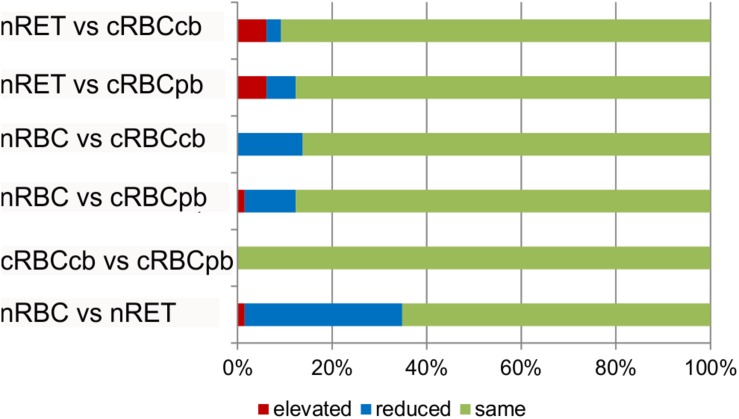
Lipid subtype analysis. Absolute amounts of phospholipid subtypes (*n* = 59) were compared between nRBCs, nRETs and cRBC^pb^ and cRBC^cb^ (*n* = 4 each). Bars show% of significantly elevated (red), reduced (blue), and unaltered (green) lipids among indicated groups.

To elucidate the statistical significance of the shown differences, subsequent Kruskal–Wallis tests with Bonferroni adjustment were performed. Phospholipid subtype analysis revealed the most significant differences between nRBCs and nRETs, with 33% reduced, 1.5% elevated and 65.5% evenly distributed lipids in nRBCs (Figure 6). Differences (>5-fold change and *p* < 0.05) were observed particularly with respect to the absolute levels of PC (PC 32:0; PC 32:1), PI (PI 38:5; PI 40:4), PE (PE 34:2; PE 40:3; PE 40:4; PE 40:5), and SM (SM 26:0; SM 26:1; SM 26:2). Interestingly, only PE 34:2 levels were significantly lower in nRETs than in nRBCs, whereas all other lipid species were significantly more abundant. Supplementary Table S4 summarizes differences between groups.

Between cRBC^pb^ and cRBC^cb^, we observed minor variations but did not find any significant differences (Figure 6). PI (PI 38:3), PE (PE 38:3; PE 40:3; PE 40:4; PE 40:5), and PC (PC 38:3) levels were significantly higher in cRBC^cb^ than in nRBCs. Furthermore, cRBC^pb^ had significantly higher levels of PI (PI 38:4) than did nRBCs (Supplementary Table S4). These higher levels may be due to the lower maturity level of cRBCs compared to nRBCs. Similar to in nRETs, PE34:2 was the only PE subclass that was lower in cRBC^pb^ than in nRBCs.

cRBCs were specifically distinct from nRETs in LPC, PE, SM and PS contents. cRBC^pb^ had significantly elevated LPC (LPC16:0; LPC 18:0; LPC 18:1) and SM (SM 20:0) values, while PS (PS 40:6) and PE (PE 34:1; PE 38:6; PE 40:7) values were much lower in cRBC^pb^ than in nRETs. cRBC^cb^ differed significantly from nRETs mainly in LPC (LPC 21:0; LPC 22:0), PE (PE 22:0; PE 36:3), and SM (SM 18:0) levels. In detail, PE 36:3 and SM 18:0 were significantly higher in cRBC^cb^ than in nRETs, while the other parameters were considerably lower (Supplementary Table S4).

Again, observed non-significant variations between cRBC^pb^ and cRBC^cb^ might explain their different significance values compared to nRBCs and nRETs. When compared to nRBCs and nRETs, above mentioned lipid subtypes showed a similar pattern of enhancement or reduction in both cRBC sources, although reaching significance only in cRBC^cb^ or cRBC^pb^.

### Biomechanical Properties of cRBCs

**FIGURE 7:**
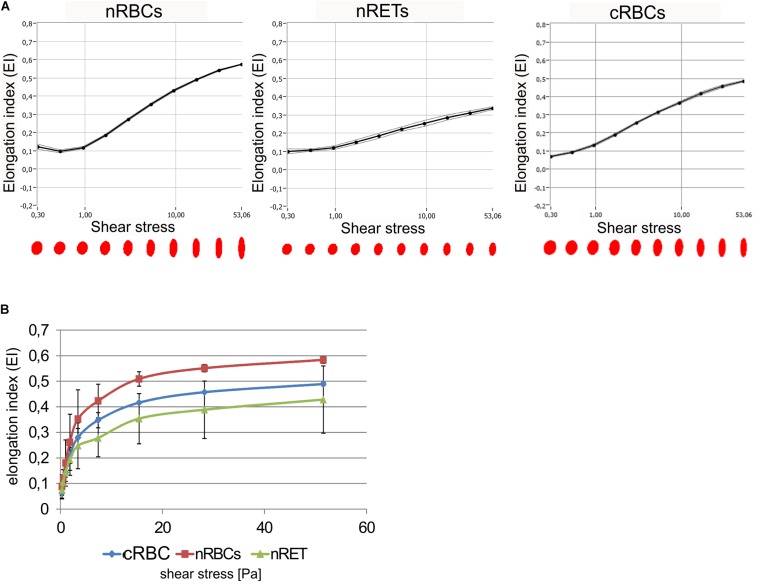
Biomechanical analysis of cRBCs. **(A)** Deformability was measured using Lorrca^®^. Deformability of cells is given as the elongation index (EI) measured under increasing shear stress. Shown are deformability curves for one representative experiment with nRBCs, cRBCs and nRETs **(B)** Deformability curves calculated from Lorrca analysis for cRBC^pb^ (*n* = 5), nRBCs (*n* = 2), and nRETs (*n* = 2) (mean ± SD). No significant differences were observed between different sources.

**FIGURE 8:**
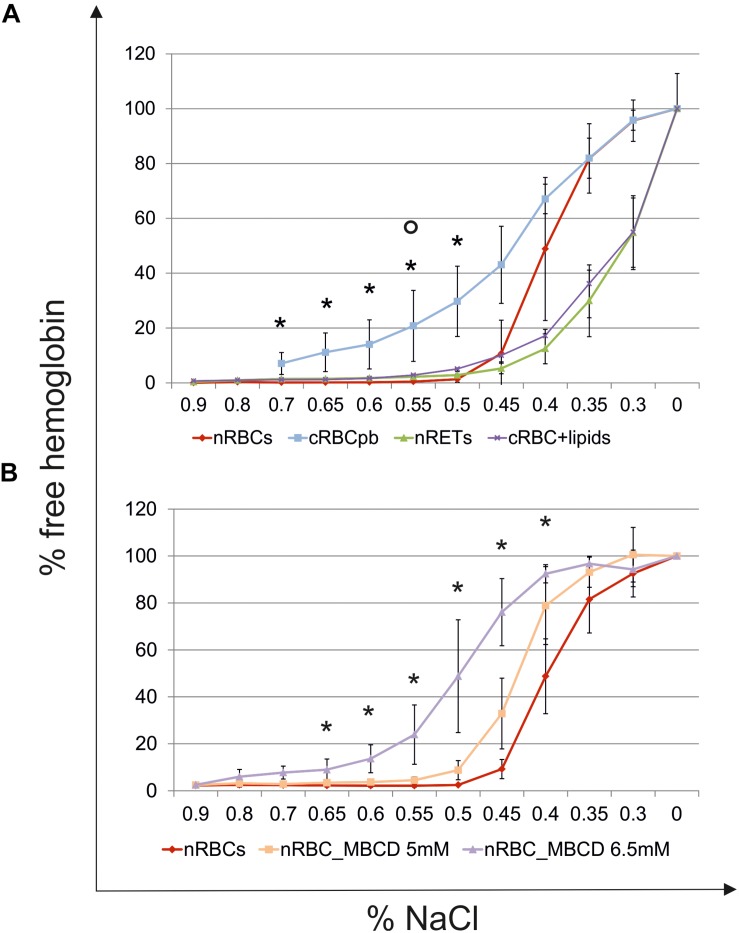
Osmotic resistance analyses. **(A)** Osmotic resistance analyses of nRBCs (*n* = 6), cRBC^pb^ (*n* = 5), nRETs (*n* = 2) and cRBC^pb^ with lipid supplementation (cRBC^pb+lipids^) (*n* = 4, mean ± SD). Osmotic resistance was calculated based on the amount of free hemoglobin after incubation of cells with decreasing NaCl concentrations (^∗^*p* < 0.05 cRBC^pb^ versus nRBCs and *p* < 0.05 cRBC^pb+lipids^ versus nRBCs). **(B)** Osmotic resistance of nRBCs after cholesterol depletion using 5 and 6.5 mM Methyl-β-cyclodextrin (MBCD). Non-treated nRBCs were used as controls (^∗^*p* < 0.05 nRBC_6.5 mM MBCD versus nRBCs).

The deformability of cRBCs was measured using a laser optical rotational cell analyzer (Lorrca^®^) (Figures 7A,B). The Elongation Index (EI) was calculated to describe the deformation of the cell in relation to the applied shear stress. Compared to the deformability curve of nRBCs (EImax 0.584), the maximum EI was lower in nRETs (EImax 0.428). The EImax of the cRBCs was positioned between these two physiological sources (EImax 0.489). Especially, in the lower range (0–10 Pa), mimicking physiological conditions, the cultured cells performed similarly to the native cells (Figure 7B). Additionally, osmotic stability assays were performed, measuring the free hemoglobin concentration after incubation of RBCs with decreasing NaCl concentrations. Evaluation of nRBCs resulted in an s-shaped curve with 50% hemolysis between 0.45 and 0.4% NaCl and a steep transition point. In contrast, analysis of nRETs revealed an s-shaped curve with 50% hemolysis between 0.35 and 0.3% NaCl (Figure 8A). The first assessment of the cRBC^pb^ resulted in a tilted s-shaped curve with increased hemolysis already at higher NaCl concentrations, while 50% hemolysis occurred between 0.45 and 0.4% NaCl. In conclusion, nRETs showed the highest stability against osmotic changes, whereas cRBCpb suffered from significantly increased hemolysis due to minimal changes in osmolality.

### Lipid Enrichment Experiments

**FIGURE 9:**
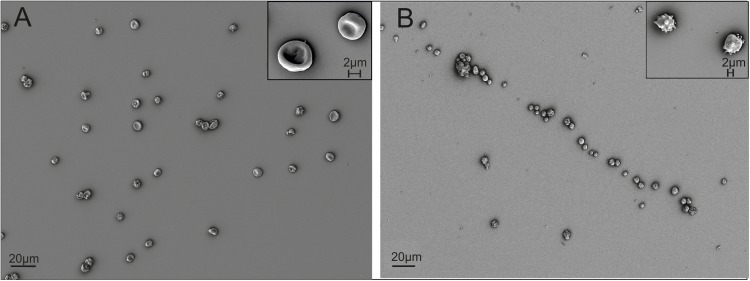
Scanning electron microscopy of cRBCs after filtration on day 18 (scale bars: 20 μm and 2 μm). **(A)** Representative picture of cRBC^pb+lipids^. **(B)** Representative picture of cRBC^pb^.

Driven by the obvious differences in the cholesterol content of cRBCs and their impaired osmotic resistance, we performed additional *ex vivo* erythropoiesis experiments from PB-derived HSCs using culture medium supplemented with cholesterol-rich lipids from d0 onward. This supplementation corresponds with concentrations of 7 mg/dl cholesterol, 5 mg/dl triglycerides and 16 mg/dl phospholipids (Supplementary Table S2). Growth and differentiation kinetics are summarized in Supplementary Figure S7. Compared to our initial experiments without lipid supplementation (Supplementary Figure S1), the differentiation of cells was slightly accelerated. Enucleated cells on day 18 showed a higher maturation grade, as indicated by New Methylene Blue staining (Figure 2) and flow cytometry analysis after Thiazole Orange and CD71 staining (Supplementary Figure S2). Compared to cRBC^pb^ the MCV was reduced (129 fl) and became more comparable to that of nRETs (Supplementary Table S3) (Nebe et al., 2011). Subsequent lipidomics analysis of enucleated day 18 cells revealed that the phospholipid composition showed marginal differences but did not change significantly compared to that of previous cultures (Figures 3, 4 and Supplementary Figure S3). However, the absolute cholesterol content of cRBC^pb^ increased from 2.3 ± 0.9 nmol/10^7^ cells to 5.0 ± 1.5 nmol/10^7^ cells after lipid supplementation (cRBC^pb+lipids^), (*p* < 0.05). Likewise, the relative cholesterol content increased from 23.7 ± 8.1% without to 40.8 ± 0.8% after lipid supplementation (Figure 3A and Supplementary Figure S3). In conclusion, the cholesterol content of cRBC^pb+lipids^ became more comparable to that of the native counterparts. Absolute cellular lipid contents with and without lipid supplementation are summarized in Figures 3B, 4. Subsequent osmotic stability analysis revealed a sigmoid curve for cRBC^pb+lipids^, analogous to that of nRETs. Consequently, the measured osmotic resistance of cRBC^pb+*lipids*^ with 50% hemolysis at 0.35–0.3% NaCl (Figure 8A) was similar to that of nRETs and indicates cholesterol as one crucial factor in membrane stability. To further confirm this hypothesis, we performed osmotic resistance measurements of nRBCs after cholesterol depletion by Methyl-β-cyclodextrin (Figure 8B; Domingues et al., 2010). Cholesterol depletion resulted in a dose dependent increase in hemolysis rates already at higher NaCl concentrations, comparable to cRBC^pb^. Scanning electron microscopy of cRBC^pb^ and cRBC^pb+lipids^ also indicated that lipid supplementation resulted in improved membrane stability, as cRBC^pb+lipids^ resembled the shape of nRETs (Figure 9A), whereas cRBC^pb^ showed mainly characteristics of echinocytes (Figure 9B). The higher resistance of cRBC^pb+lipids^ became further evident calculating the recovery of reticulocytes after filtration of cultured cells on days 18. Recovery significantly increased from a relatively low level (40.1 ± 9.7%) in cRBC^pb^ to 84.3 ± 12.5% in cRBC^pb+lipids^ (*p* < 0.01, *n* = 10).

## Discussion

*Ex vivo* culturing of RBCs is an attractive and common tool in various fields of RBC research. In addition, it is a promising approach in replacing classical blood components in clinical transfusion medicine. As a prerequisite for these applications, the integrity of the RBC membrane is of utmost importance. Otherwise, cRBCs will suffer from impaired membrane function, which results in reduced expansion and differentiation, altered biomechanical properties and finally reduced survival *ex vivo* and *in vivo*. In addition to the cytoskeleton and embedded proteins, the lipid bilayer is critical for membrane integrity. Current knowledge about lipid synthesis and the changes in membrane lipid composition during erythropoiesis is scarce. Although cRBCs suffer from increased fragility and reduced survival, studies investigating the lipid content of cRBCs are still lacking.

The present study compared the lipid composition of cRBCs with that of their native counterparts, nRETs and nRBCs. In line with published data, the *ex vivo* erythropoiesis protocol (Giarratana et al., 2011; Betz et al., 2016) allowed for the homogenous maturation of HSCs into terminally differentiated erythroid cells. Nucleic acid staining and analysis of cell surface marker expression showed that the cRBCs were in a transient maturation stage between spheroid nRETs and biconcave-shaped mature erythrocytes, accounting for their still high plasma membrane content. Likewise, lipidomics data revealed a total lipid content of cRBCs between that of the two native counterparts. In nRETs, similar to their higher membrane amount, the lipid content was higher than in nRBCs. In our study, the source of human HSPCs (PB or CB) did not make any difference in the lipid content and composition of cRBCs. Comparable observations were made by Wilson et al. (2016) for proteomics data.

High-resolution mass spectrometry analysis revealed most disparities in lipid composition between nRBCs and nRETs, mostly reflecting the membrane remodeling process during RBC maturation, resulting in reorganization and loss of 20% of the membrane area (Liu et al., 2010; Minetti et al., 2018). Although lower in absolute amount, we found exactly the same proportional distribution of cholesterol and phospholipids in nRBCs and nRETs. In both sources, cholesterol represented the largest fraction, with 50% of the lipid content. In cRBCs, the most obvious discrepancy with the native counterparts was observed in the percentage of cellular cholesterol, which turned out to be due to culture-based malnutrition. In contrast, observed minor differences in the amount of various phospholipids might be explained by their intermediate stage of maturation, associated with ongoing changes in the plasma membrane content.

With respect to the results of Buchwald et al. (2000) that with increasing cholesterol content, membrane flexibility decreases, the membrane characteristics of nRBCs and nRETs were expected to be similar in stiffness, with clear differences from less rigid cRBCs. This was not found for Lorrca^®^ testing, where cRBCs performed comparably to nRBCs and nRETs. In contrast, cRBCs suffered from reduced osmotic resistance by showing enhanced hemolysis due to minimal osmotic changes. The osmotic resistance is known to be affected by membrane integrity as well as by the surface-area-to-volume ratio (Fischbach and Dunning, 2009). To investigate these likely culture-related effects, further *ex vivo* erythropoiesis experiments were performed. By adding cholesterol-rich lipids to the standard culture medium, the concentration of cholesterol was enhanced from 3 mg/dl to a relatively high level of 7 mg/dl. As expected, cRBCs from lipid-enriched cultures showed higher relative cholesterol content, near the content of native cells, whereas no significant changes in phospholipid content were observed compared to those of previous cRBCs. Consequently, cRBCs with lipid supplementation recovered and showed osmotic resistance comparable to that of the nRETs. Likewise, cholesterol depletion of nRBCs by Methyl-β-cyclodextrin treatment resulted in reduced osmotic resistance comparable to cRBC^pb^ without supplementation. These results demonstrate that lipid uptake, especially of cholesterol, from the medium is crucial for the membrane functionality of cRBCs, and therefore sufficient lipid supplementation is mandatory during *ex vivo* erythropoiesis.

The necessity of cholesterol for membrane biosynthesis and survival of RBCs is also evidenced *in vivo*, where mutations in genes of cholesterol synthesis lead to anemias (Jira et al., 2003; Wang et al., 2014). Our findings are further in line with those of Leberbauer et al. (2005) who reported enhanced erythroid expansion due to the addition of cholesterol-rich lipids to the culture medium and a more recent publication by the group of Migliaccio, who stated that *ex vivo* expansion of immature erythroblasts is highly dependent on plasma lipoproteins (Zingariello et al., 2019). Both groups examined the impact of steroids like dexamethasone and lipid supplementation on the *ex vivo* expansion and maturation of cRBCs without investigating the final lipid composition of cRBCs (Zingariello et al., 2019). In our study, expansion rates were not massively affected by lipid supplementation, while a tendency toward faster differentiation was noticed.

Despite statistical significance, the differences found in phospholipid subtype analysis were rather marginal and might primarily be explained by the intermediate maturity level of cRBCs. This is further in line with the observation that RBCs are able to undergo *de novo* lipid synthesis, deacetylation and reacetylation, as well as exchange with other membranes and the environment during their maturation from nRETs to nRBCs (Soupene et al., 2008; Soupene and Kuypers, 2012; Wu et al., 2016). Phospholipids are mostly synthesized by the Kennedy pathway, a *de novo* pathway in the Golgi and endoplasmic reticulum (ER), and repaired by a remodeling pathway called Lands’ cycle (Moessinger et al., 2014). The final organization of phospholipids occurs after the enucleation and release of the nRETs into the blood stream for terminal maturation. Due to a lack of Golgi apparatus and ER, mature nRBCs do not have *de novo* synthesis of phospholipids (Wu et al., 2016).

Investigating the phospholipids and their subtypes in more detail, we found in the immature nRETs and cRBCs the most prominent increases in PC, SM, PE, PI, and LPC compared to these levels in nRBCs. First, we focused on components of the outer leaflet. The diacyl PC content, particularly PC 32:0, was found to be the lowest in nRBCs. This result might explain the higher deformability of erythrocytes, as these molecules have a cylinder-like molecular shape and can consequently pack neatly in a planar bilayer. In a simplified view, the rigidity of membranes results from the packing gradient of its constituent molecules (proteins and lipids) at different depths within the bilayer. Lipids yield different molecular shapes (cylinders, cones, inverted-cones) depending on the composition of their hydrophilic heads and hydrophobic tails; when forced into a planar membrane environment, these shapes experience varying lateral packing densities, which may either increase or decrease the membrane’s resistance to deformation (Frolov et al., 2011). Furthermore, SM subclasses were decreased in nRBCs compared to those in cRBCs and nRETs. SM also has a cylinder-like molecular shape but packs even more tightly than PC due to strong intermolecular hydrogen bonding (Slotte, 2016). Thus, the higher the SM content is, the stiffer the membrane. Taken together, our results obtained for the outer membrane lipids are in line with the literature, stating the highest deformability for nRBCs.

In the inner leaflet, we found a decrease in PE content in nRBCs compared to that in nRETs. A high PE content is known to augment membrane stiffness by both intermolecular hydrogen bonding (Boggs, 1980) and significant hydrocarbon splay compared to the small head group (Kollmitzer et al., 2013). Consequently, the lower PE content of the more flexible nRBCs is plausible. Interestingly, PE 34:2 was significantly lower in cRBCs and nRETs, whereas the levels of PE subclasses with longer and more unsaturated hydrocarbons were increased. The resulting membrane bending resistance may be compensated for by the decrease in PC and SM in nRBCs. Alternatively, the observed increase in the number of double bonds, as shown in the Supplementary Material in PE, may increase the overall membrane fluidity and hence contribute to increased cell flexibility. Furthermore, nRBCs showed a proportional decrease in the PI content compared to all of the less-mature cell types. Only the more unsaturated species, such as PI 38:4 and PI 38:5, remained elevated, speculatively to maintain necessary cellular function and PIP/PLC signaling. Phosphoinositides are important regulators of autophagy by controlling the cytosol-membrane interface (Schink et al., 2013). These findings are in line with ongoing autophagic and membrane restructuring processes during terminal transition from the reticulocyte to the erythrocyte (Mankelow et al., 2015).

In summary, the obtained results demonstrate the importance of the lipid bilayer composition on the functionality on cRBCs. Cholesterol-deficient cRBCs suffer from impaired osmotic resistance. This observation might be the main reason for the observed fragility of cRBCs and their reduced survival rates *ex vivo* and *in vivo*. *Ex vivo* culturing of RBCs is a common tool in all fields of RBC research. In the absence of a scientifically based rationale, current lipid supplementation underlies a high variability and is generally below the concentrations reported in the present study. The maintenance of RBC membrane integrity by lipid supplementation will improve results obtained from *ex vivo*-generated RBCs due to a decrease in hemolysis and an improvement of biomechanical properties. In our study this was demonstrated by improved shape of cultured cells as well as higher recovery after filtration. This further strengthens the efforts to make *ex vivo*-generated RBCs clinically applicable in terms of their storability and *in vivo* survival, especially for the blood supply of patients with rare blood groups or alloimmunization against multiple blood group antigens. To further elucidate the role of lipids membrane stability of cRBCs, detailed analyses of the impact of single lipoprotein fractions for lipid supplementation as well as their optimal source, concentration and administration time are necessary.

## Data Availability Statement

The datasets generated for this study are available on request to the corresponding author.

## Ethics Statement

The study was reviewed and approved by the Ethics Committee of the Medical University of Graz, Graz, Austria. The patients/participants provided their written informed consent to participate in this study.

## Author Contributions

CB, PS, and ID conceptualized and designed the study. CB, ID, and KS were involved in collection and assembly of the data. MT did mass spectrometry analyses. DK contributed the scanning electron microscopy. CB, HK, GP, ST, GH, PS, and ID contributed to the data analysis and interpretation. CB, ID, HK, and GP wrote the manuscript. PS edited the manuscript. All authors edited and revised the manuscript and gave final approval for publication.

## Conflict of Interest

The authors declare that the research was conducted in the absence of any commercial or financial relationships that could be construed as a potential conflict of interest.
