# Supplementary material for: Cholesterol Deficiency Causes Impaired Osmotic Stability of Cultured Red Blood Cells
Source: Front Physiol. 2019 Dec 20;10:1529. doi: 10.3389/fphys.2019.01529 (PMC6933518; doi:10.3389/fphys.2019.01529)
Supplement: Supplementary file 1 [file Data_Sheet_1.docx]

Supplementary Material

**Dorn et al.,** Cholesterol deficiency causes impaired osmotic stability of cultured red blood cells

1. **Supplementary Methods**

**Erythroid differentiation**

Isolated CD34+ HSPCs from PB and CB were cultured in an established three-step differentiation model ([Giarratana et al., 2011](#_ENREF_8);[Betz et al., 2016](#_ENREF_2)) in Iscove’s liquid medium (Biochrom) with 5% human plasma (Octapharma), 10 µg/ml insulin (Sigma Aldrich) and 330 µg/ml human holo-transferrin (Spicac). For the lipid enrichment experiments, the medium was supplemented with 4 mg/dl cholesterol-rich lipids (L4646; Sigma Aldrich) from day 0 onwards. Cells were stimulated with 100 ng/ml stem cell factor (SCF), 5 ng/ml interleukin-3 (IL-3) (both from Peprotech), 3 U/ml erythropoietin (EPO) (Erypo, Janssen Biologics B.V) and 10^-6^M hydrocortisone (Sigma-Aldrich) on days 0-8; 100 ng/ml SCF and 3 U/ml EPO on days 8-11; and 3 U/ml EPO until day 18. Erythroid differentiation was monitored by flow cytometry of cell surface markers and morphologic evaluation of cytospins stained with May-Gruenwald-Giemsa (Hemafix™, Biomed). Hemoglobin expression was visualized by costaining with neutral benzidine (o-dianisidine, Sigma-Aldrich). At least 300 cells were enumerated per slide. For flow cytometry analyses, 1x10^5^ cells were incubated for 20 min at RT with respective antibodies against CD45, CD71 (Becton Dickinson), CD36 and glycophorin A (GPA) (both Beckman Coulter). Cells were costained with 4’,6-diamidino-2-phenylindole (DAPI) (Invitrogen, Thermo Fisher Scientific) to exclude dead cells and extruded nuclei. Measurement was performed on a Navios™ flow cytometer (Beckman Coulter). For determination of remaining nucleic acids, Thiazole Orange staining after filtration was done using Retic Count^TM^ (Becton Dickinson), following the manufacturer’s instructions. Cells were analyzed on a CytoFLEX flow cytometer (Beckman Coulter).

**Mass spectrometry analysis of cholesterol and phospholipids**

Filtered, enucleated day 18 cells (1×10^7^) were prepared for mass spectrometry analyses. The following lipids were analyzed: Cholesterol, PC (32:0, 32:1, 34:0, 34:1, 34:2, 36:1, 36:2, 36:3, 36:4, 38:3, 38:4, 38:5, 38:6), PE (22:0, 26:0, 34:1, 34:2, 36:1, 36:2, 36:3, 36:4, 38:3, 38:4, 38:5, 38:6, 40:3, 40:4, 40:5, 40:6, 40:7), PS (38:4, 40:6), LPC (16:0, 17:0, 18:0, 18:1, 20:4, 21:0, 22:0), SM (14:0, 16:0, 16:1, 18:0, 20:0, 22:0, 22:1, 23:0, 24:0, 24:1, 24:2, 26:0, 26:1, 26:2) and PI (36:4, 38:3, 38:4, 38:5, 40:3, 40:4). Cells were washed twice in PBS, and dry pellets were resuspended in 1 ml of 70% methanol to prevent oxidation and stored at -80°C until analysis. Lipids from all sources (nRBCs, nRETs, cRBC^pb^, cRBC^cb^ and cRBC^pb+lipids^) were extracted with an MTBE protocol according to Matyash et al.([Matyash et al., 2008](#_ENREF_14)). Lipid extracts were suspended in 500 µl of CHCl_3_ / MeOH (1:1), and 12:0/13:0 PC, 17:0/20:4 PC, 14:1/17:0 PC, 21:0/22:6 PC, 17:1 LPC, 17:1 LPE, SP-Mix LM6002 (1.5 µM each), 12:0/13:0 PE, 17:0/20:4 PE, 14:1/17:0 PE, 21:0/22:6 PE (2.5 µM each), 12:0/13:0 PS, 17:0/20:4 PS, 14:1/17:0 PS, 21:0/22:6 PS (3 µM each), 12:0/13:0 PI, 17:0/20:4 PI, 14:1/17:0 PI, 21:0/22:6 PI (2 µM each) and cholesterol-D7 standard (80 µM) were added as internal standards. Two microliters were injected on a Waters BEH C8, 100 x 1 mm, 1.7 µm HPLC column used with an Ultimate 3000 UHPLC (Thermo Scientific). Solvent A was water with 1% ammonia acetate and 0.1% formic acid, and solvent B was acetonitrile/2-propanol (5:2) with 1% ammonia acetate and 0.1% formic acid. Gradient elution started at 50% mobile phase B, rising to 100% B over 40 min; 100% B was held for 10 min, and the column was re-equilibrated with 50% B for 8 min before the next injection. The flow rate was 150 µl/min and the samples were kept at 8°C. The Orbitrap Velos Pro hybrid mass spectrometer was operated in data-dependent acquisition mode using a HESI II ion source. The ion source parameters for positive polarity were as follows: source voltage: 4.5 kV; source temperature: 275^°^C; sheath gas: 25 arbitrary units; aux gas: 9 arbitrary units; sweep gas: 0 arbitrary units; and capillary temperature: 300°C. The ion source parameters for negative ion mode were as follows: source voltage: 3.8 kV; source temperature: 325°C; sheath gas 30 arbitrary units; aux gas: 10 arbitrary units; sweep gas: 0 arbitrary units; and capillary temperature: 300°C. The automatic gain control target value was set to 10^6^ ions to enter the mass analyzer, with a maximum ion accumulation time of 500 ms.

**Scanning electron microscopy**

Cells were mounted on coverslips, fixed in 1% glutaraldehyde in 0.1 M phosphate buffer pH 7.4 for 1 h at room temperature and postfixed with 1% Osmiumtetroxid for 1h at room temperature. Samples were subsequently dehydrated in graded series of ethanol (30%, 50%, 70%, 80%, 90%, 96%, and absolute ethanol). Furthermore, critical point drying (Baltec CPD 30) and sputtercoating (Baltec Sputter Coater 500) was done. Coverslips were placed on stubs covered with a conductive double coated carbon tape. Images were taken using a Sigma 500VP FE-SEM with a SE Detector (Zeiss, Oberkochen, Germany) operated at an acceleration voltage of 3 kV.

1. **Supplementary Results**

**Phospholipid chain lengths and double bounds**

Besides phospholipid content, average chain lengths (Figure S4) and double bonds (Figure S5) of the phospholipid classes were analyzed between the groups. Compared to nRETs, nRBCs showed significant differences in PI chain length, PE double bonds and LPC double bonds (p<0.05). Comparison of weighted chain length and double bonds revealed some significant differences between native sources and cRBCs. nRBCs showed significant differences from cRBC^cb^ but not cRBC^pb^ in overall double bonds, PI double bonds and PE chain length (p<0.05). Interestingly, nRETs revealed more significant variations to cRBCs. PS chain length and double bonds revealed significant differences to nRETs in both cRBC^cb^ and cRBC^pb^ respectively (p<0.05). Furthermore, LPC chain length and PI double bonds varied significantly between nRETs and cRBC^cb^. Analyses revealed further significantly different values of PC chain length, PC double bonds and SM double bonds between nRETs and cRBC^pb^ (p<0.05). The two cRBC groups differed only in overall chain length (p<0.05). Interestingly, the biggest variations were found between nRET and cRBCpb. The possible physiological impact of observed differences has to be elucidated in further studies.

1. **Supplementary Figures and Tables**

**3.1 Supplementary Figures**

**
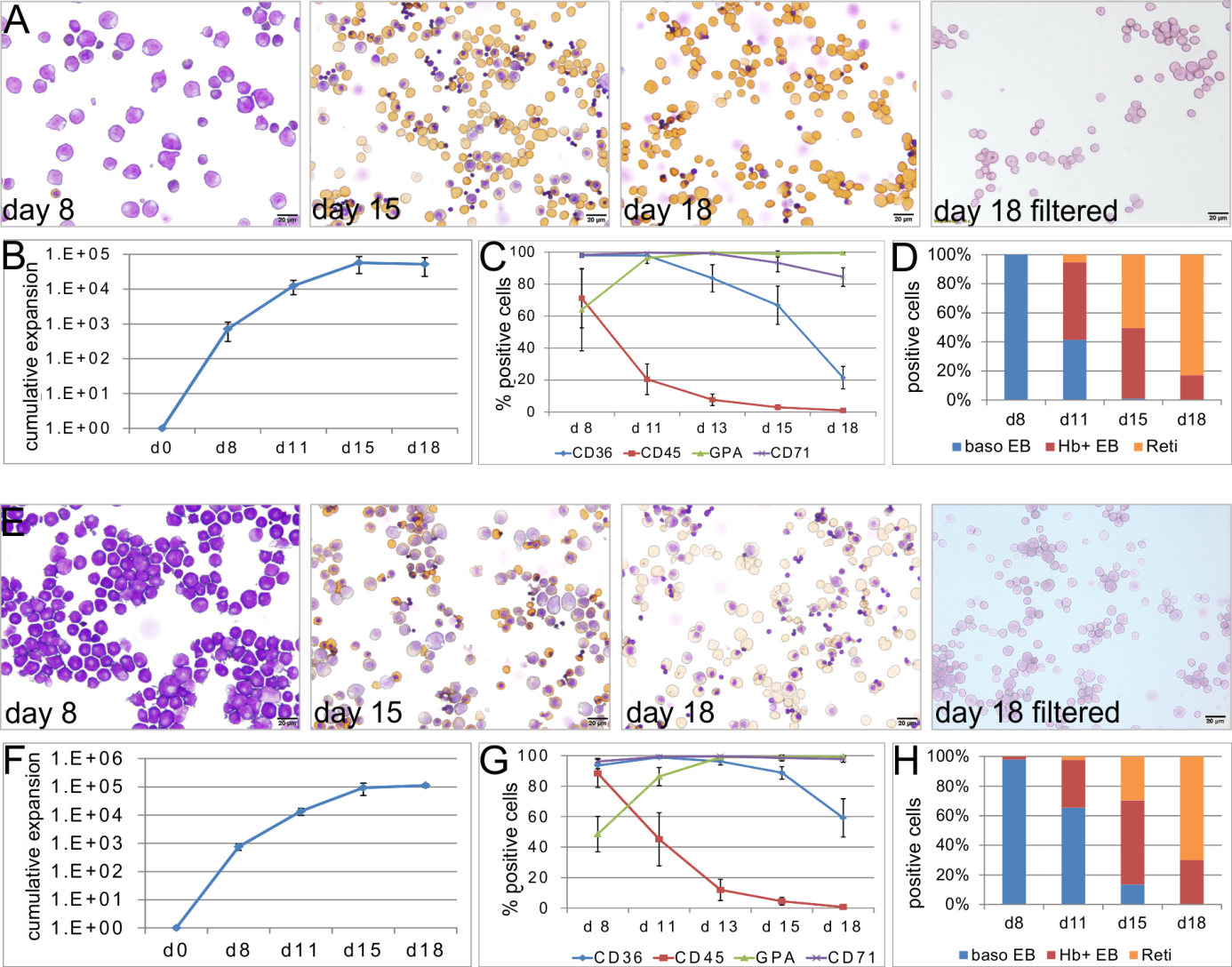
**

**Supplementary Figure 1**: Erythroid differentiation of PB-derived CD34^+^ HSPCs (n=4) and CB-derived CD34^+^ HSPCs (n=4) in the described three-phase liquid assay over 18 days (mean ± SD). Figures A-D represent PB-derived erythropoiesis, and Figures E-H CB-derived erythropoiesis. **A and E:** Representative pictures of May-Gruenwald-Giemsa and neutral benzidine costained cytospins from days 8, 15 and 18 of ex-vivo erythropoiesis from PB- (A) and CB-derived HSPCs (**E**) and enucleated cells after filtration. **B and F:** Cumulative expansion days 0-18 of erythropoiesis. **C and G:** Cell surface marker expression measured by flow cytometry **D and H:** Differential count of stained cytospin samples, showing the mean amounts of basophilic erythroblasts (baso EB), hemoglobin-positive erythroblasts (Hb^+^EB) and enucleated reticulocytes (Reti).


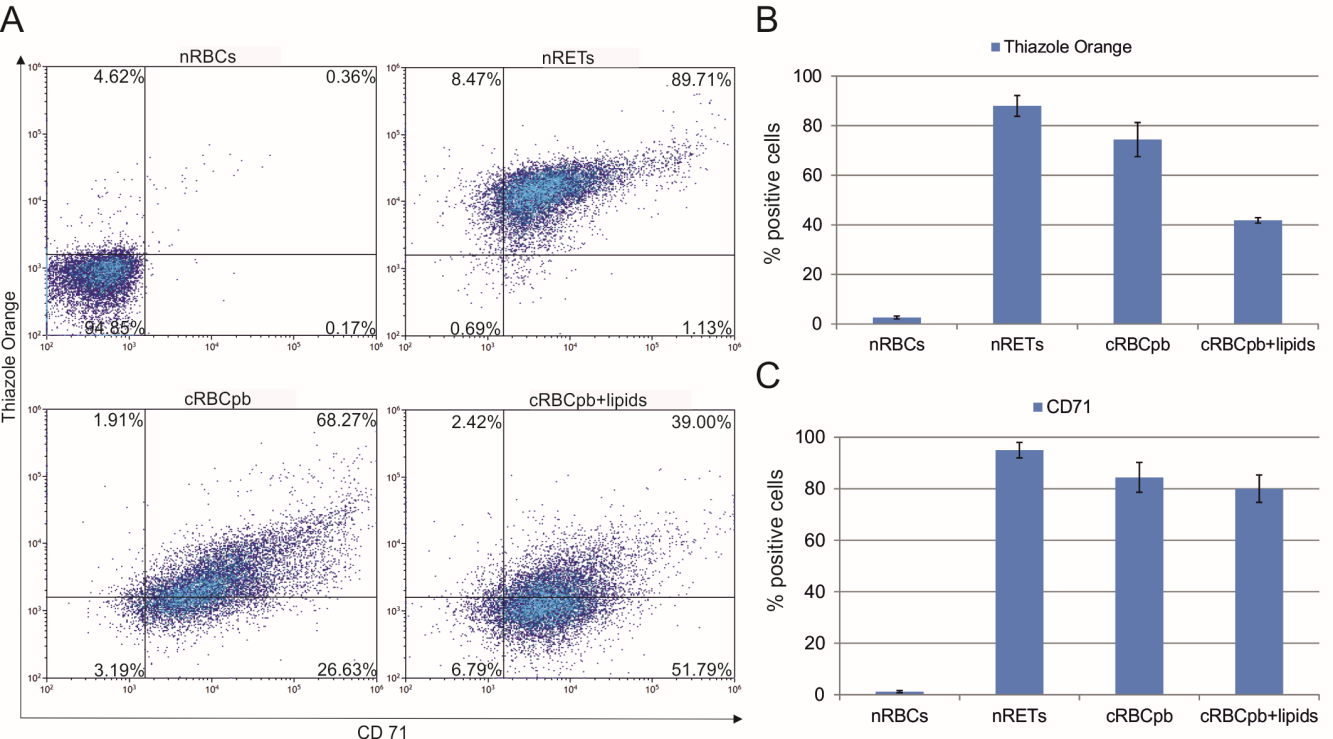


**Supplementary Figure 2**: Maturation stage of nRBCs, nRETs, cRBC^pb^ and cRBC^pb+lipids^ (day 18, after filtration) determined by flow cytometry after Thiazole Orange and CD71 staining. **A:** Representative flow cytometry pictures from all sources. **B:** Percentages of Thiazole Orange positive cells. Bars represent mean ± SD, n=4. **C:** Percentages of CD71^+^ cells. Bars represent mean ± SD, n=6.


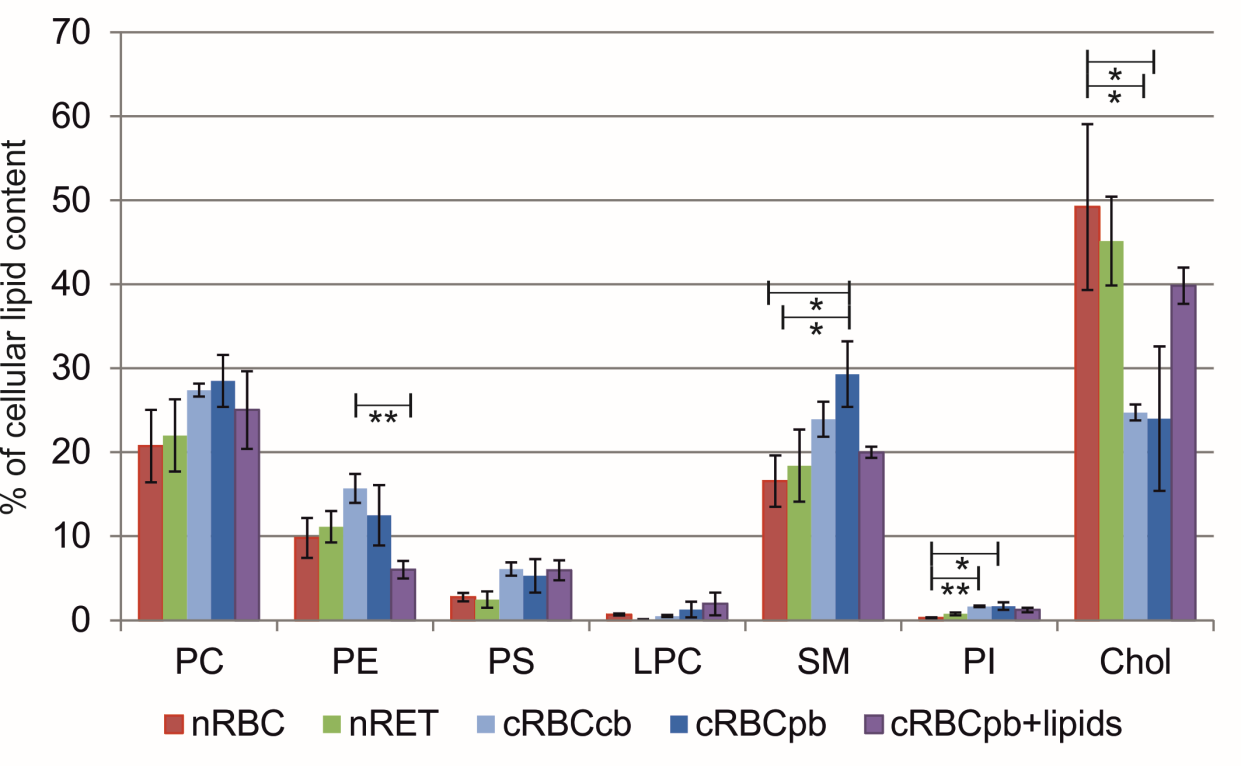


**Supplementary Figure 3:** Proportional lipid content measured by high-resolution mass spectrometry in nRBCs, nRETs, cRBC^cb^, cRBC^pb^ and cRBC after lipid supplementation (cRBC^pb+lipids^), (n=4 each). Bars represent the proportional lipid content of PC, PE, PS, LPC, SM, PI and cholesterol (mean ± SD) *p<0.05, **p<0.01.


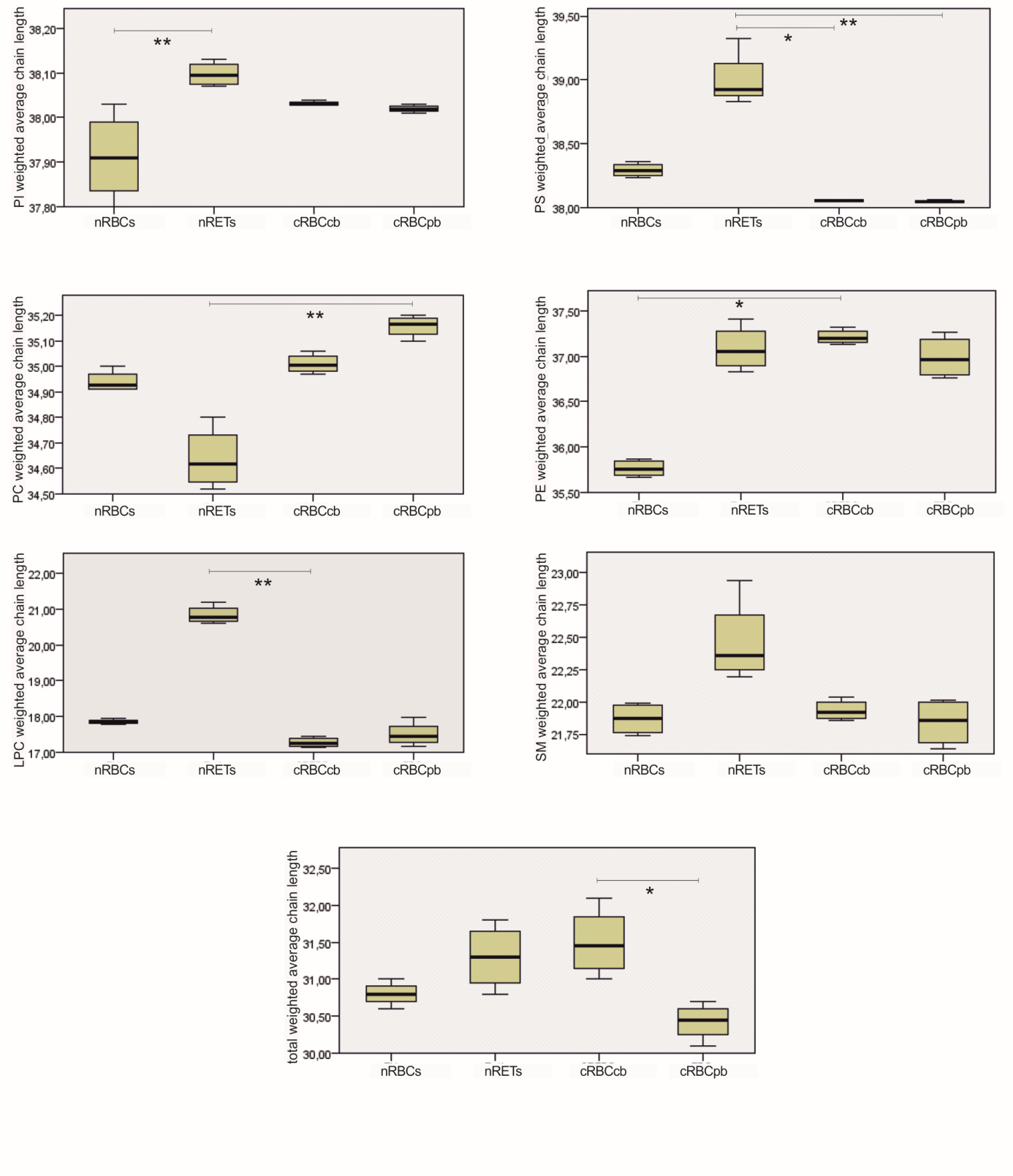


**Supplementary Figure 4:** Average weighted chain length of total lipid content and measured lipid species in nRBCs, nRETs, cRBC^pb^ and cRBC^cb^, each n=4. Samples were analyzed with Kruskal-Wallis Test for independent samples. Significance values were adjusted with Bonferroni correction (*p<0.05, **p<0.01).


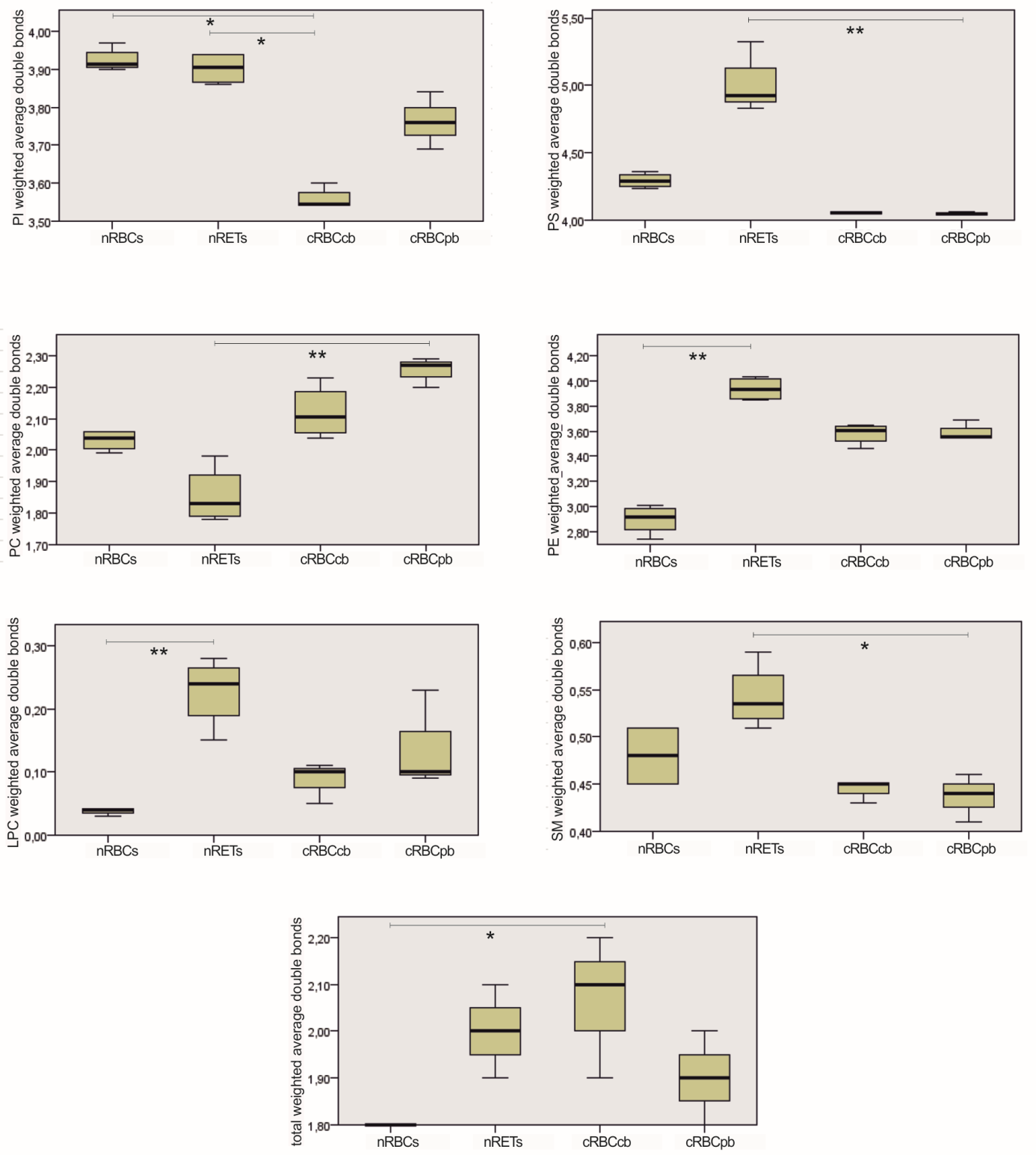


**Supplementary Figure 5:** Average weighted double bonds of total lipid content and measured lipid species in nRBCs, nRETs, cRBC^pb^ and cRBC^cb^, each n=4. Samples were analyzed with Kruskal-Wallis Test for independent samples. Significance values were adjusted with Bonferroni correction (*p<0.05, **p<0.01).

**
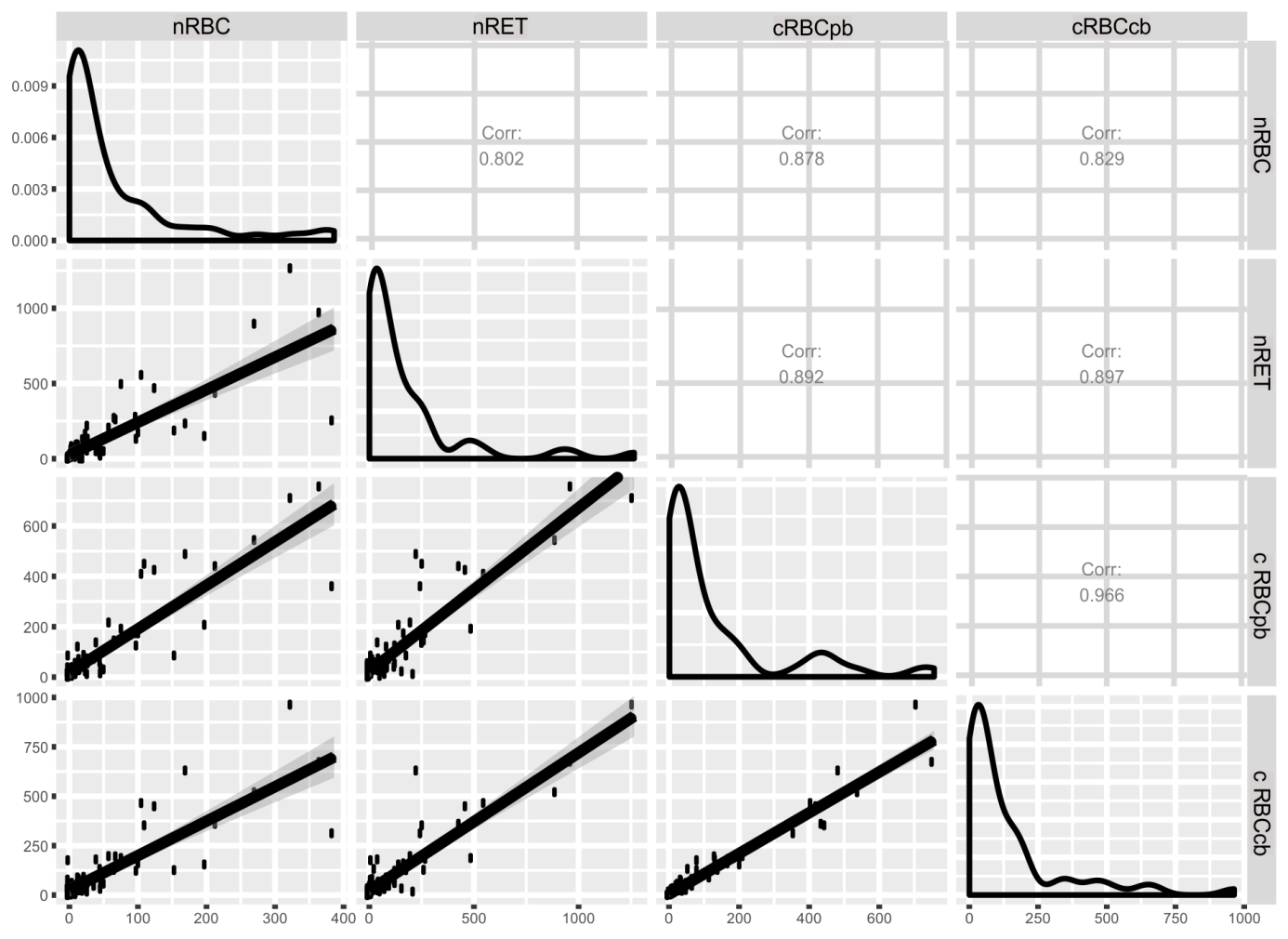
**

**Supplementary Figure 6:** Correlation analysis for values obtained from phospholipid subtype analysis. Pearson correlation between nRBCs, nRETs, cRBC^pb^ and cRBC^cb^ was calculated using R statistical program Version 3.5.1 extended with “GGally” library.


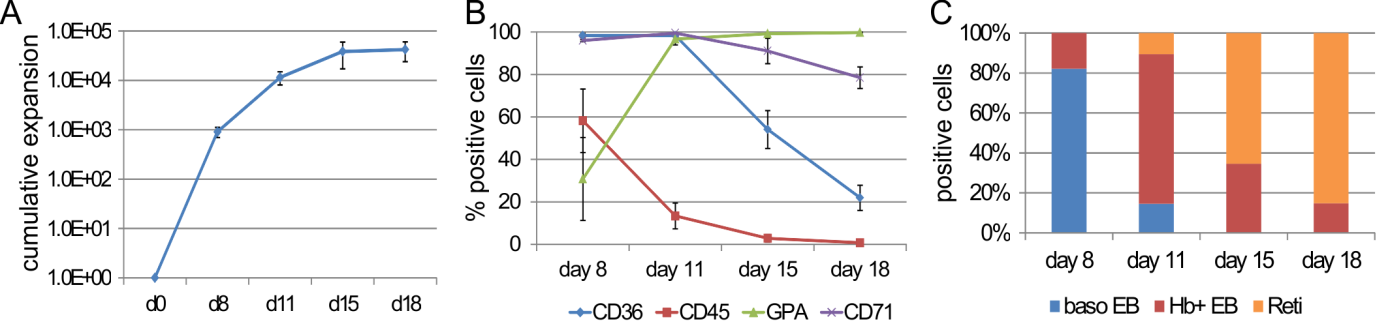


**Supplementary Figure 7:** Erythroid differentiation of PB-derived HSPCs in the three-phase liquid culture after additional lipid supplementation (cRBC^pb+lipids^) days 0-18 (n=6). **A:** Cumulative expansion of erythroid cells **B:** Cell surface marker expression measured by flow cytometry **C:** Differential count of stained cytospin samples showing % of basophilic erythroblasts (basoEB), hemoglobin-positive erythroblasts (Hb^+^EB) and enucleated reticulocytes (Reti).

**3.2 Supplementary Tables**

**Table S1:** Overview of published ex vivo erythropoiesis systems, used medium, addition of plasma or serum as potential lipid sources (bovine serum albumin (BSA), human serum albumin (HSA), fetal bovine serum (FBS)), and additional supplementation of lipids as far as indicated in the original publications, (n.g. not given).

| **Reference** | **Medium** | **Plasma or serum as potential lipid source** | **Lipid supplements** |
| --- | --- | --- | --- |
|  |  |  |  |
| ([Couch et al., 2019](#_ENREF_5)) | IMDM | 5% human plasma |  |
| ([Kikuchi et al., 2018](#_ENREF_12)) | IMDM | 3% human plasma  2% FBS | - |
| ([Huang et al., 2017](#_ENREF_11)) | IMDM StemSpan® | 2% human plasma  3% human serum | - |
| ([Trakarnsanga et al., 2017](#_ENREF_22)) | IMDM StemSpan® | 3% human serum 2% FBS | - |
| ([Hu et al., 2013](#_ENREF_10)) | IMDM | 2% human plasma 3% human serum | - |
| ([Griffiths et al., 2012](#_ENREF_9)) | IMDM | 1% BSA  2% human plasma | 1 µl/ml cholesterol rich lipids |
| ([Giarratana et al., 2011](#_ENREF_8)) | IMDM | 5% human plasma | - |
| ([Timmins et al., 2011](#_ENREF_21)) | IMDM | 1% BSA | - |
| ([Migliaccio et al., 2010](#_ENREF_16)) | IMDM | 10% HSA | 1.2 mg/ml soy bean lecithin 400 µg/ml cholesterol (egg yolk) |
| ([van den Akker et al., 2010](#_ENREF_23)) | Stemspan® | 3% human plasma | 40 µg/ml cholesterol-rich lipids |
| ([Baek et al., 2009](#_ENREF_1)) | IMDM | 1% HSA 5% human plasma | 2 µg/ml cholesterol |
| ([Boehm et al., 2009](#_ENREF_3)) | IMDM | 1% BSA 10% FBS | - |
| ([Fujimi et al., 2008](#_ENREF_6)) | X-VIVO10 IMDM | 1% BSA 2% human serum 0.5% Plasmanate | - |
| ([Cheung et al., 2007](#_ENREF_4)) | StemBio A® | - | - |
| ([Miharada et al., 2006](#_ENREF_17)) | StemSpan® H300 | 5% Plasmanate® Cutter | 4ng/ml linoleic acid 200 ng/ml cholesterol |
| ([Giarratana et al., 2005](#_ENREF_7)) | IMDM | 1% BSA | - |
| ([Leberbauer et al., 2005](#_ENREF_13)) | StemSpan® | - | 40 µg/ml cholesterol-rich lipids |
| ([Migliaccio et al., 2002](#_ENREF_15)) | IMDM | 20% FBS | - |
| ([Neildez-Nguyen et al., 2002](#_ENREF_19)) | n.g. | 2% BSA | 30 µg/ml soy bean lecithin 7.5 µg/ml cholesterol |

**Table S2:** Content of cholesterol, triglycerides and phospholipids (mg/dl) in human plasma (Octapharma) and cholesterol-rich lipids (Sigma-Aldrich L4646) and final concentrations in the culture media.

|  |  |  | cRBC^pb^, cRBC^cb^ | cRBC^pb+lipids^ |
| --- | --- | --- | --- | --- |
|  | human plasma | cholesterol-rich lipids | culture medium, 5% plasma | culture medium,  5% plasma,  40 µg/ml cholesterol-rich lipids |
| cholesterol | 59 | 830 | 3 | 7 |
| triglycerides | 58 | 15 | 5 | 5 |
| phospholipids | 154 | 503 | 13 | 16 |

**Table S3: A:** Mean corpuscular volume MCV [fl], mean corpuscular hemoglobin content (CHm) [pg] and mean corpuscular hemoglobin concentration (CHCm) [g/dl] of cRBC^pb^ (n=12), cRBC^pb + lipids^ (n=6), nRBC^pb^ (n=45), nRET^pb^ (n=30), nRBC^cb^ (n=30) and nRET^cb^ (n=30) assessed by ADVIA analyzer. **B:** Published reference values for nRBCs and nRETs measured by ADVIA analyzer ([Orkin S., 2009](#_ENREF_20);[Nebe T, 2011](#_ENREF_18)).

| **A** | **MCV (fl)** | **CHm (pg)** | **CHCm (g/dl)** |
| --- | --- | --- | --- |
| cRBC^pb^ (n=12)  cRBC^pb+lipids^ (n=6) | 141.5 ± 9.7  128.7 ± 11.7 | 35.9 ± 7.6  32.7 ± 1.3 | 25.5 ± 6.0  26.0 ± 2.2 |
| nRBC^pb^ (n=45)  nRET^pb^ (n=30) | 89.3 ± 1.5  103.5 ± 3.0 | 30.8 ± 1.5  32.8 ± 1.4 | 34.7 + 1.5  31.7 ± 1.1 |
| nRBC^cb^ (n=30)  nRET^cb^ (n=30) | 107.7 ± 5.9  125.1 ± 5.7 | 31 ± 1.5  35.2 ± 1 | 35.3 ± 1.3  28.3 ± 1.1 |

| **B** | **MCV (fl)** | **CHm (pg)** | **CHCm (g/dl)** |
| --- | --- | --- | --- |
| nRBC^pb^ ([Nebe T, 2011](#_ENREF_18)) | 80.1 - 95.3 | 27 - 33.2 | 32.6 - 37.2 |
| nRET^pb^ ([Nebe T, 2011](#_ENREF_18))  nRET^cb^ ([Orkin S., 2009](#_ENREF_20)) | 92 - 120  125 ± 8.6 | 28 - 35  32.9 ± 3.1 | 27 - 33  27.1 + 1.95 |

**Table S4**: Differences in the absolute amount of phospholipid subtypes measured by mass spectrometry among nRBCs, nRETs and cRBCcb and cRBCpb (p<0.05 was considered statistically significant). Given are the absolute amount of lipids in pmol/10^7^ cells (mean ± SD), the ratio between the sources and the p-values. Shown are only parameters that revealed significant differences. For comparison, each line also includes concentrations of lipids in the other cell types (ns not significant)

1. **Supplementary References**

Baek, E.J., Kim, H.S., Kim, J.H., Kim, N.J., and Kim, H.O. (2009). Stroma-free mass production of clinical-grade red blood cells (RBCs) by using poloxamer 188 as an RBC survival enhancer. *Transfusion* 49**,** 2285-2295.

Betz, J., Dorn, I., Kouzel, I.U., Bauwens, A., Meisen, I., Kemper, B., Bielaszewska, M., Mormann, M., Weymann, L., Sibrowski, W., Karch, H., Schlenke, P., and Muthing, J. (2016). Shiga toxin of enterohaemorrhagic Escherichia coli directly injures developing human erythrocytes. *Cell Microbiol* 18**,** 1339-1348.

Boehm, D., Murphy, W.G., and Al-Rubeai, M. (2009). The potential of human peripheral blood derived CD34+ cells for ex vivo red blood cell production. *J Biotechnol* 144**,** 127-134.

Cheung, J.O., Casals-Pascual, C., Roberts, D.J., and Watt, S.M. (2007). A small-scale serum-free liquid cell culture model of erythropoiesis to assess the effects of exogenous factors. *J Immunol Methods* 319**,** 104-117.

Couch, T., Murphy, Z., Getman, M., Kurita, R., Nakamura, Y., and Steiner, L.A. (2019). Human erythroblasts with c-Kit activating mutations have reduced cell culture costs and remain capable of terminal maturation. *Exp Hematol* 74**,** 19-24 e14.

Fujimi, A., Matsunaga, T., Kobune, M., Kawano, Y., Nagaya, T., Tanaka, I., Iyama, S., Hayashi, T., Sato, T., Miyanishi, K., Sagawa, T., Sato, Y., Takimoto, R., Takayama, T., Kato, J., Gasa, S., Sakai, H., Tsuchida, E., Ikebuchi, K., Hamada, H., and Niitsu, Y. (2008). Ex vivo large-scale generation of human red blood cells from cord blood CD34+ cells by co-culturing with macrophages. *Int J Hematol* 87**,** 339-350.

Giarratana, M.C., Kobari, L., Lapillonne, H., Chalmers, D., Kiger, L., Cynober, T., Marden, M.C., Wajcman, H., and Douay, L. (2005). Ex vivo generation of fully mature human red blood cells from hematopoietic stem cells. *Nat Biotechnol* 23**,** 69-74.

Giarratana, M.C., Rouard, H., Dumont, A., Kiger, L., Safeukui, I., Le Pennec, P.Y., Francois, S., Trugnan, G., Peyrard, T., Marie, T., Jolly, S., Hebert, N., Mazurier, C., Mario, N., Harmand, L., Lapillonne, H., Devaux, J.Y., and Douay, L. (2011). Proof of principle for transfusion of in vitro-generated red blood cells. *Blood* 118**,** 5071-5079.

Griffiths, R.E., Kupzig, S., Cogan, N., Mankelow, T.J., Betin, V.M., Trakarnsanga, K., Massey, E.J., Lane, J.D., Parsons, S.F., and Anstee, D.J. (2012). Maturing reticulocytes internalize plasma membrane in glycophorin A-containing vesicles that fuse with autophagosomes before exocytosis. *Blood* 119**,** 6296-6306.

Hu, J., Liu, J., Xue, F., Halverson, G., Reid, M., Guo, A., Chen, L., Raza, A., Galili, N., Jaffray, J., Lane, J., Chasis, J.A., Taylor, N., Mohandas, N., and An, X. (2013). Isolation and functional characterization of human erythroblasts at distinct stages: implications for understanding of normal and disordered erythropoiesis in vivo. *Blood* 121**,** 3246-3253.

Huang, N.J., Pishesha, N., Mukherjee, J., Zhang, S., Deshycka, R., Sudaryo, V., Dong, M., Shoemaker, C.B., and Lodish, H.F. (2017). Genetically engineered red cells expressing single domain camelid antibodies confer long-term protection against botulinum neurotoxin. *Nat Commun* 8**,** 423.

Kikuchi, G., Kurita, R., Ogasawara, K., Isa, K., Tsuneyama, H., Nakamura, Y., Yabe, R., Shiba, M., Tadokoro, K., Nagai, T., and Satake, M. (2018). Application of immortalized human erythroid progenitor cell line in serologic tests to detect red blood cell alloantibodies. *Transfusion* 58**,** 2675-2682.

Leberbauer, C., Boulme, F., Unfried, G., Huber, J., Beug, H., and Mullner, E.W. (2005). Different steroids co-regulate long-term expansion versus terminal differentiation in primary human erythroid progenitors. *Blood* 105**,** 85-94.

Matyash, V., Liebisch, G., Kurzchalia, T.V., Shevchenko, A., and Schwudke, D. (2008). Lipid extraction by methyl-tert-butyl ether for high-throughput lipidomics. *J Lipid Res* 49**,** 1137-1146.

Migliaccio, G., Di Pietro, R., Di Giacomo, V., Di Baldassarre, A., Migliaccio, A.R., Maccioni, L., Galanello, R., and Papayannopoulou, T. (2002). In vitro mass production of human erythroid cells from the blood of normal donors and of thalassemic patients. *Blood Cells Mol Dis* 28**,** 169-180.

Migliaccio, G., Sanchez, M., Masiello, F., Tirelli, V., Varricchio, L., Whitsett, C., and Migliaccio, A.R. (2010). Humanized culture medium for clinical expansion of human erythroblasts. *Cell Transplant* 19**,** 453-469.

Miharada, K., Hiroyama, T., Sudo, K., Nagasawa, T., and Nakamura, Y. (2006). Efficient enucleation of erythroblasts differentiated in vitro from hematopoietic stem and progenitor cells. *Nat Biotechnol* 24**,** 1255-1256.

Nebe T, B.F., Bruegel M, Fiedler Gm, Gutensohn K, Heimpel H, Krebs N, Ossendorf M, Schuff-Werner P, Stamminger G, Baum J (2011). Multicentric determination of reference ranges for automated blood counts. *J Lab Med* 35**,** 1-25.

Neildez-Nguyen, T.M., Wajcman, H., Marden, M.C., Bensidhoum, M., Moncollin, V., Giarratana, M.C., Kobari, L., Thierry, D., and Douay, L. (2002). Human erythroid cells produced ex vivo at large scale differentiate into red blood cells in vivo. *Nat Biotechnol* 20**,** 467-472.

Orkin S., N.D. (2009). *Nathan's and Orkin's Hematology of Infancy and Childhood.* Saunders Elsevier.

Timmins, N.E., Athanasas, S., Gunther, M., Buntine, P., and Nielsen, L.K. (2011). Ultra-high-yield manufacture of red blood cells from hematopoietic stem cells. *Tissue Eng Part C Methods* 17**,** 1131-1137.

Trakarnsanga, K., Griffiths, R.E., Wilson, M.C., Blair, A., Satchwell, T.J., Meinders, M., Cogan, N., Kupzig, S., Kurita, R., Nakamura, Y., Toye, A.M., Anstee, D.J., and Frayne, J. (2017). An immortalized adult human erythroid line facilitates sustainable and scalable generation of functional red cells. *Nat Commun* 8**,** 14750.

Van Den Akker, E., Satchwell, T.J., Pellegrin, S., Daniels, G., and Toye, A.M. (2010). The majority of the in vitro erythroid expansion potential resides in CD34(-) cells, outweighing the contribution of CD34(+) cells and significantly increasing the erythroblast yield from peripheral blood samples. *Haematologica* 95**,** 1594-1598.
